# Neighborhood disparities in stroke and myocardial infarction mortality: a GIS and spatial scan statistics approach

**DOI:** 10.1186/1471-2458-11-644

**Published:** 2011-08-12

**Authors:** Ashley Pedigo, Tim Aldrich, Agricola Odoi

**Affiliations:** 1 The University of Tennessee, Department of Comparative Medicine, 2407 River Drive Knoxville, Tennessee 37996-4543, USA; 2 East Tennessee State University, Department of Biostatistics and Epidemiology, P.O. Box 70259, Johnson City, TN 37614-1709, USA

## Abstract

**Background:**

Stroke and myocardial infarction (MI) are serious public health burdens in the US. These burdens vary by geographic location with the highest mortality risks reported in the southeastern US. While these disparities have been investigated at state and county levels, little is known regarding disparities in risk at lower levels of geography, such as neighborhoods. Therefore, the objective of this study was to investigate spatial patterns of stroke and MI mortality risks in the East Tennessee Appalachian Region so as to identify neighborhoods with the highest risks.

**Methods:**

Stroke and MI mortality data for the period 1999-2007, obtained free of charge upon request from the Tennessee Department of Health, were aggregated to the census tract (neighborhood) level. Mortality risks were age-standardized by the direct method. To adjust for spatial autocorrelation, population heterogeneity, and variance instability, standardized risks were smoothed using Spatial Empirical Bayesian technique. Spatial clusters of high risks were identified using spatial scan statistics, with a discrete Poisson model adjusted for age and using a 5% scanning window. Significance testing was performed using 999 Monte Carlo permutations. Logistic models were used to investigate neighborhood level socioeconomic and demographic predictors of the identified spatial clusters.

**Results:**

There were 3,824 stroke deaths and 5,018 MI deaths. Neighborhoods with significantly high mortality risks were identified. Annual stroke mortality risks ranged from 0 to 182 per 100,000 population (median: 55.6), while annual MI mortality risks ranged from 0 to 243 per 100,000 population (median: 65.5). Stroke and MI mortality risks exceeded the state risks of 67.5 and 85.5 in 28% and 32% of the neighborhoods, respectively. Six and ten significant (p < 0.001) spatial clusters of high risk of stroke and MI mortality were identified, respectively. Neighborhoods belonging to high risk clusters of stroke and MI mortality tended to have high proportions of the population with low education attainment.

**Conclusions:**

These methods for identifying disparities in mortality risks across neighborhoods are useful for identifying high risk communities and for guiding population health programs aimed at addressing health disparities and improving population health.

## Background

On average, every 34 and 40 seconds, myocardial infarction (MI) and stroke events occur in the US, respectively [1]. Stroke ranks third in causes of death and is the leading cause of debilitation among Americans [2]. It is estimated that approximately 15% of those who have an MI will die of it [1]. These health conditions are serious economic burdens to the US health system with annual costs estimated at $73.7 billion for stroke and $177.1 billion for MI [1].

Place of residence is an important determinant of cardiovascular health and disparities in the burdens of stroke and MI have been observed for different geographic areas [1-3]. The highest risks of mortality have been reported in the southeastern US [1,4-6] and in populations living in rural areas [7-9], particularly in the Appalachian region [10,11]. Many areas of the Appalachian region, including parts of Tennessee, form a portion of the US "stroke belt". Tennessee ranks 3^rd ^highest in the US for stroke [1], and had an annual age-adjusted stroke mortality risk for the period 2000-2006 of 67.5 deaths per 100,000 persons compared to the national risk of 53.5 deaths per 100,000 persons [12]. For coronary heart disease including MI, Tennessee ranks 4^th ^highest in the US [1] with an annual age-adjusted mortality risk for the period 2000-2006 of 85.5 deaths per 100,000 persons compared to the national risk of 58.9 death per 100,000 persons [12]

The geographic distributions of stroke and MI mortality have been investigated at state and county levels [1,5,11]. However, geographic disparities have been shown to exist even after adjusting for variations in common risk factors like demographic factors (race, age), socioeconomic measures (income, education), behaviors (smoking, physical activity), and other conditions (diabetes, hypertension) [4,10,11,13]. These findings suggest that geographic variation in stroke and MI mortality could be due to more localized distributions of neighborhood risk factors. The clustering of determinants of stroke and MI at the neighborhood level can greatly affect the planning, implementation, and focus of health initiatives that seek to reduce disparities. Therefore, research should focus on identifying disparities at the neighborhood level to better understand health needs and thus, provide needs-based health services [3,14]. While many studies have defined neighborhoods as census tracts or smaller geographic units, the neighborhoods have not been used as the unit of analysis for many past studies investigating cardiovascular disease and stroke [15-21]. Rather, these studies have investigated neighborhood characteristics as contextual effects in multilevel models that seek to explain individual level risk. Thus, ecological studies are needed to investigate the spatial patterns and clustering of high mortality risk with the neighborhood as the unit of analysis since this is important in identifying high risk communities and targeting resources to address health disparities and improve population health at the local level.

When investigating disease patterns in small geographic areas like neighborhoods, however, there are some challenges that must be addressed. Due to population heterogeneity, mortality risks from areas of low population will likely have higher variances and therefore be more unstable than those from areas of high population [22]. This variance instability of small geographic areas is referred to as the small number problem [23]. Spatial smoothing of risks is used to mitigate this issue by reducing the "noise" from areas with low population and therefore high variances [24].

With these issues in mind, the objective of this study was to investigate spatial patterns and detect local neighborhood clusters of high risk of stroke and MI mortality in the East Tennessee Appalachian Region. The identification of neighborhoods with high risks is expected to aid local health planners in understanding the specific neighborhood health needs to guide health planning and provision of health services. Thus, identified clusters of high risks of stroke and MI mortality will be useful in guiding resource allocation, service provision, and policy decisions at the local/neighborhood level that are crucial for addressing neighborhood health disparities.

## Methods

### Study area and data collection

The study area included eleven counties of the East Tennessee Appalachian Region that have some of the highest risks of stroke and/or MI in the state: Claiborne, Cocke, Grainger, Greene, Hamblen, Hancock, Hawkins, Jefferson, Knox, Sevier, and Union counties. This area had a population of just over 780,000 persons in 2000 and included 168 census tracts. Census tracts (CTs) are statistical subdivisions of a county that have between 2,500 and 8,000 persons, do not cross county boundaries, and are homogenous with respect to population characteristics, economic status, and living conditions [25]. Since they are good proxies of natural neighborhood boundaries and are therefore useful in describing neighborhood population characteristics and health disparities [26,27], CTs were chosen as the geographical unit of analysis and were used to represent neighborhoods in this study.

Mortality data from 1999 to 2007 were obtained free of charge, upon request, from the Tennessee Department of Health. Thus, although these data are freely available on request from the responsible authorities, they are not currently openly available for internet downloads. Stroke and MI deaths were identified by ICD 10 codes I60-I69 and I21-I22, respectively. For the 8,842 mortality records obtained, complete street address data were available for 94%, while the other 6% had missing or inadequate (such as post office box) address data. The addresses were geo-coded using BatchGeo [28], an online geo-coding service which implements the Google Maps geocoding application programming interface (API) that has some of the highest quality geocoding databases available [29,30]. Exact, or roof top, address matches were obtained for 67% of the data, while 30% were range interpolated between two points on the street and 3% were matched to the zipcode. The geographic coordinates were imported into ArcGIS 9.3 [31] where point-in-polygon join was used to link the mortality data to the openly available census tract level cartographic boundary files downloaded from the U.S. Census Bureau website [32].

Census tract level socioeconomic, demographic, and population data for the study area were obtained from the openly available census 2000 summary file 3 [33]. Since these data are available in the US only through the decennial census, the 2000 data was deemed best suited to match the disease data (1999-2007). The neighborhood variables chosen to be assessed as potential predictors of the geographic distribution of MI and stroke high risk mortality clusters were based on current knowledge in the literature. They include: black race [3,5,8,34], gender [2,8,35,36], age 65 years and older [2,8,15,37], household income [15,16,18,21,38], education less than high school [8,21,39,40], population below poverty [16,21,41], median housing value [39,42,43], geography (urban versus rural) [3,7,8,44], and factors like employment, single parent families, marital status, and housing ownership that have been used in composite measures of socioeconomic status (SES) or deprivation [39,42,43,45].

### Data analysis

#### Data management

One neighborhood in Knox county, that had a population of 232 and included a mental health facility, was removed from the analysis due to missing data values for most of the variables. With the exception of median household income, median housing value, and family size, all variables were analyzed as the proportion of the population in each CT (neighborhood).

#### Descriptive analyses, risk standardization and spatial smoothing

All descriptive analyses were done in SAS 9.2 [46]. Significance of the difference in median age between genders was assessed using the Wilcoxon rank sum test [47]. Mortality risks for neighborhoods were age-adjusted using direct standardization in Stata 11 [48]. All risks were expressed as the annual number of deaths per 100,000 population.

The raw (unsmoothed) age-adjusted risks were expected to have high variances due to the small number problem since there were areas of low population and some neighborhoods with only a few cases of stroke/MI in the study area [23]. To address this issue, as well as adjust for spatial autocorrelation and population heterogeneity, the raw age-adjusted risks were smoothed using Spatial Empirical Bayes (SEB) smoothing using 2^nd ^order queen weights in GeoDa [49]. In this smoothing method, the risks for low population neighborhoods in areas without clear spatial patterns are shrunk toward the global mean of the study area [22,50]. Conversely, in areas where obvious spatial patterns exist, the less reliable estimates from low population areas are adjusted towards a local mean. Thus, the SEB smoothed risks are more stable than raw (unsmoothed) risks [24].

#### Detection and identification of stroke and MI clusters

To detect the presence of high risk stroke and MI clusters and identify their locations, the spatial scan statistic, implemented in SaTScan, was used [51]. The technique uses circular windows of variable radius that move across the study area to compare the number of deaths in the window with what would be expected if the deaths were distributed randomly in space [51]. The window radius varies from zero up to a specified maximum. Each window defines a set of different neighboring CTs, such that if the geographic centroid of a CT is contained in the window, then the deaths and population from that whole CT are included. Clusters are identified based on a likelihood ratio test [52] with a p-value obtained using Monte Carlo replications [53]. The primary cluster, with the highest significant likelihood, is interpreted such that there is an increased risk of stroke/MI mortality within the window compared to outside [54].

Non-overlapping, spatial clusters of high risk of stroke/MI mortality were identified using a purely spatial, discrete Poisson model [52] adjusted for age distribution. Since the results of this analysis can be sensitive to model parameters, particularly window size, care must be taken in its choice. The goal of the current analyses was to identify local clusters of high mortality risks among neighborhoods. Thus, similar to another study [55], the window size of 5% of the total population was chosen based on the population of the largest neighborhood so that potentially one single neighborhood could constitute a distinct high risk cluster.

#### Logistic modeling of predictors of high risk stroke or MI clusters

The outcome of interest in this modeling was binary, reflecting whether a neighborhood belonged to a cluster or not. Univariate associations of continuous variables with the outcomes were assessed using Wilcoxon rank sum test for non-normally distributed data, while chi-square and exact tests were used for categorical variables. Variables with significant associations based on a liberal p-value (p = 0.20) were considered in the modeling process along with some non-significant variables that had been shown in literature to be strongly associated with the outcome.

Multiple logistic models were used to investigate potential associations between log odds of a neighborhood being in a high risk stroke or MI cluster and a number of neighborhood level socioeconomic and demographic characteristics. The assumption of linearity of continuous variables with the log odds of the outcome (belonging to a stroke or MI cluster) for logistic modeling were assessed using graphical methods. Only the proportions of the population ≥ 65 years and of single parent families met this assumption for stroke cluster, while the proportions of population with less than high school education, those living below poverty and median housing value met the assumption for the MI outcome. Therefore, these variables were modeled as continuous variables. The variables not meeting the linearity assumption were transformed into categorical variables using either *a priori *considerations or quartile cutpoints from the distribution of the variable.

The model was built by starting with the full model and then removing variables based on the following criteria: (1) the highest non-significant p-value (with significance set to p = 0.05); (2) a likelihood ratio test of the model with and without the variable that was non-significant; and (3) the variable was not an important confounder of other variables in the model. Variables were considered important confounders if their removal from the model resulted in a large (greater than 20%) change in the coefficients of any of the remaining variables in the model. Categorical variables were analyzed as regular dummy variables. The significance in the model of each group of dummy variables (belonging to one categorical variable) was analyzed using a likelihood ratio test. Two-way interaction terms between gender, race, age, income, education, poverty, and geography were assessed for statistical significance [8,35,45,56]. Model fits were assessed using the Pearson and Hosmer-Lemeshow goodness of fit tests and residual diagnostics. The predictive abilities of the models were evaluated using sensitivity, specificity, and overall correct classifications.

### Cartographic displays

All cartographic manipulations and displays were done in ArcGIS 9.3 [31]. The intervals for displaying the age-adjusted SEB smoothed mortality risks of stroke and MI in the choropleth maps were determined using Jenk's optimization classification scheme. Since SEB risks are more appropriate for mapping in small areas compared to unsmoothed risks [23,24], only the former are presented. Significant spatial clusters were displayed in ArcGIS 9.3 [31].

## Results

### Description of stroke and MI deaths

There were 3,824 stroke deaths in the study area from 1999 to 2007. No stroke deaths were reported in 18 of the 168 neighborhoods. Women accounted for 2,435 (63.7%) of the stroke deaths. The median age was significantly (p < 0.001) lower for men (median 78; range 4-103), than women (median 81; range 3-103). Persons dying from stroke or MI in the study were primarily white (94%) and had less than a high school education (45%). It is worth noting that 92% of the population in the study area was white, while 25% of the population older than 18 years had less than high school education.

Myocardial infarction was the cause of 5,018 deaths during the study period. No deaths were reported in 17 neighborhoods; 15 of these neighborhoods also had no reported stroke deaths. More MI deaths occurred in men (2,745 deaths, 54.6%) than women (45.4%). Again, the median age of death was significantly (p < 0.001) lower for men (median 71; range 21-102), than women (median 81; 27-106).

### Spatial distribution of mortality risks

#### Stroke risks

**Figure 1:**
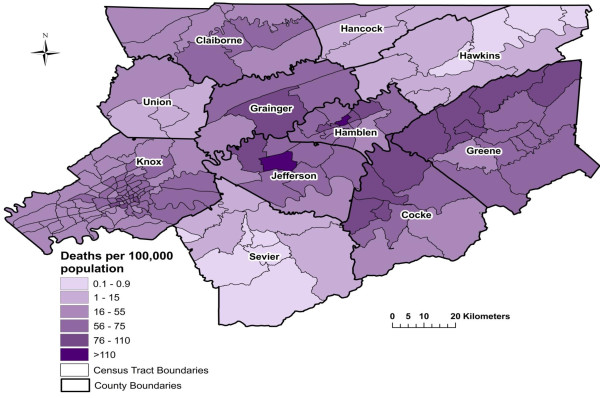
**Spatial Empirical Bayes smoothed age-adjusted stroke mortality risk per 100,000 population from 1999 to 2007 in East Tennessee Appalachian region**.

The annual median age-adjusted raw (unsmoothed) stroke risk for the study area was 55.6 deaths/100,000 population (range: 0-182), with 28% of the neighborhoods exceeding the state stroke mortality risk of 67.5 [12]. Similarly, the annual median SEB smoothed stroke risk was 56.1 deaths/100,000 population (range: 0.1-174). The annual median risk for the study area remained constant from 1999 to 2007. The highest stroke risks (greater than 110 deaths/100,000) were observed in three neighborhoods in Knox county and one neighborhood each in Jefferson and Hamblen counties (Figure 1). It appeared that the neighborhoods with stroke risks higher than the state risk were concentrated across neighborhoods in the northwest portions of Cocke and Greene counties, in addition to a few neighborhoods in Grainger, Hamblen, and Jefferson counties, as well as in the downtown area of Knox county. These neighborhoods are primarily located in or near city centers in the study area.

#### Myocardial infarction risks

**Figure 2:**
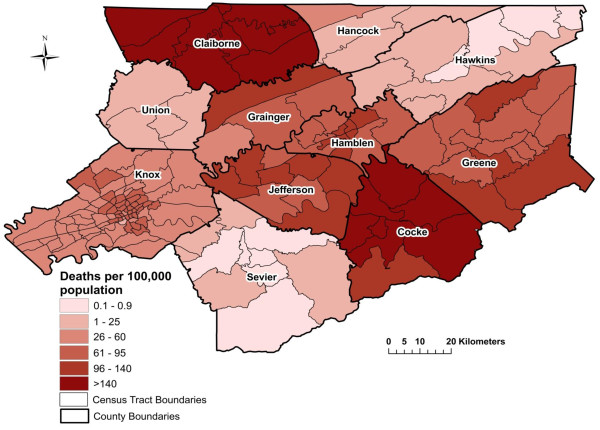
**Spatial Empirical Bayes smoothed age-adjusted myocardial infarction mortality risk per 100,000 population from 1999 to 2007 in East Tennessee Appalachian region**.

The annual median raw (unsmoothed) age-adjusted MI mortality risk was 65.5 deaths/100,000 population (range: 0-243), while the median SEB smoothed risk was 63.5 (range: 0.5-235). Myocardial infarction mortality risks in the study area were higher than the state risk of 85.5 [12] in 32% of the neighborhoods. The spatial distribution of neighborhood risks revealed patterns of high risks across the study area (Figure 2). The areas with the highest MI risks (greater than 140 deaths/100,000) included all neighborhoods in Claiborne county and all but one neighborhood in Cocke county. In addition to these counties, neighborhoods with risks above the state risk were also located in Greene, Jefferson, Hamblen, Grainger, and Knox counties in a pattern very similar to that for stroke risks.

### Spatial clusters of high stroke/MI mortality risks

**Figure 3:**
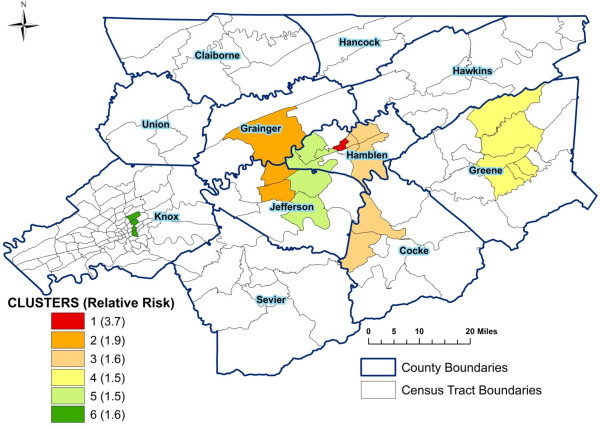
**Significant spatial clusters of high age-adjusted stroke mortality risks from 1999 to 2007 in East Tennessee Appalachian region**.

**Figure 4:**
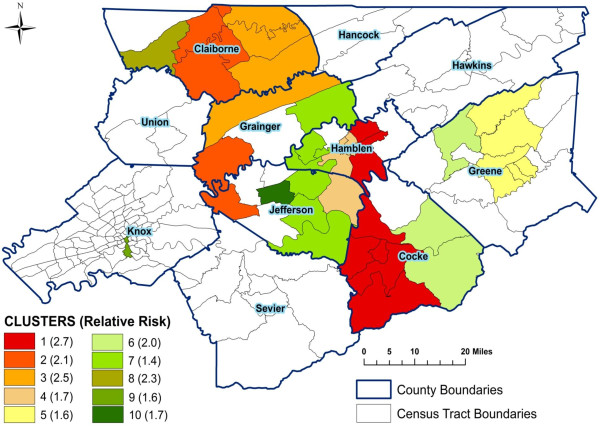
**Significant spatial clusters of high age-adjusted myocardial infarction mortality risks from 1999 to 2007 in East Tennessee Appalachian region**.

Table 1 displays results of identified significant spatial stroke and MI mortality clusters. For each cluster, the table gives the number of census tracts in the cluster, the total population, the observed number of stroke or MI deaths in the cluster area, the expected number of deaths based on the Poisson model, the estimated annual number of cases per 100,000 persons, and the significance level (p-value) obtained from the likelihood ratio test with Monte Carlo permutations. Figures 3 and 4 display geographic distributions of the significant spatial clusters of stroke and MI, respectively.

**Table 1:** Spatial clusters of age-adjusted stroke and myocardial infarction mortality risks from 1999 to 2007 in East Tennessee Appalachian region

Cluster	# of Census Tracts (Neighborhoods)	Population	Observed # of Deaths	Expected # of Deaths	Annual # of Deaths/100,000 Persons	P-value
**Stroke**						
1	1	5,447	136	37.76	195.6	0.001
2	3	17,243	174	91.95	102.8	0.001
3	6	34,887	270	174.24	84.2	0.001
4	6	30,158	266	187.63	77.0	0.001
5	5	24,711	180	120.65	81.0	0.001
6	4	12,008	107	67.27	86.4	0.004
**Myocardial Infarction**						
1	7	36,945	608	243.77	177.7	0.001
2	6	24,596	334	159.80	148.9	0.001
3	4	13,856	213	88.78	171.0	0.001
4	6	28,823	333	197.74	120.0	0.001
5	6	30,158	363	236.98	109.2	0.001
6	3	9,568	124	61.61	143.4	0.001
7	7	35,548	325	231.94	99.9	0.001
8	1	2,818	47	20.94	160.0	0.001
9	4	8,566	88	54.98	114.1	0.009

#: Number.

#### Stroke clusters

Six significant (p < 0.001) spatial clusters of high risk of stroke mortality were identified (Table 1 and Figure 3). The smallest cluster, which was also the primary cluster, was comprised of only 1 neighborhood in Hamblen county. The relative risk of this cluster was 3.7 (Figure 3), implying that the risk of death from stroke was 3.7 times higher within cluster 1 than other neighborhoods in the study area. Relative risks for the secondary clusters ranged from 1.5 to 1.9. Cluster 3 accounted for the highest number of stroke deaths and was composed of 6 neighborhoods in Cocke and Hamblen counties. The second largest cluster (cluster 4) included 6 neighborhoods in Greene county. The majority of the high risk stroke clusters were located in or near city centers.

#### Myocardial infarction clusters

There were nine significant (p < 0.009) spatial clusters of high risk of MI mortality (Table 1 and Figure 4). The primary cluster was the largest cluster in both the number of MI deaths and geographic size, and included neighborhoods in Cocke and Hamblen counties. The populations in cluster 1 neighborhoods had a risk of death from MI that was 2.7 times greater than other neighborhoods in the study area. Relative risks for the secondary clusters ranged from 1.4 to 2.5. Cluster 7 was the second largest and included neighborhoods in Jefferson, Hamblen, and Grainger counties. Neighborhoods in Claiborne, Greene, and Knox counties were also parts of significant high risk MI clusters. The majority (76%) of neighborhoods in significant high risk stroke clusters also belonged to significant high risk MI clusters.

### Predictors of high risk stroke and myocardial infarction spatial clusters

#### Stroke

The univariate associations of the socioeconomic and demographic variables of interest with the outcome of belonging to a high risk stroke cluster are presented in Table 2. Variables with significant associations, based on a liberal p-value = 0.20 were further assessed in the multivariable logistic model. Even though gender was non-significant it was included because disparities in stroke risk and mortality by gender have been reported in literature [2,8,40,41]. The other non-significant variables were not included because they were each highly correlated (r > 0.70) with median household income. The final model had a highly significant (p = 0.0002) likelihood. The proportion of the population with less than a high school education (p = 0.015) and that were black (p = 0.019) were significant variables in the model (Table 3). Neighborhood geography (rural, suburban, urban) was not significant (p = 0.1), but was included in the final model because it was an important confounder of race such that its removal resulted in a 30% change for coefficients for proportion of blacks. No interaction terms were significant at the p < 0.05 level. Neighborhoods with higher proportion of population with less than a high school education had significantly higher odds of belonging to a stroke cluster compared to those with low proportion of the population with less than high school education.

**Table 2:** Univariate associations of high risk stroke mortality clusters with neighborhood socioeconomic and demographic factors

Neighborhood level socioeconomic and demographic variables	Significance value	
	Stroke cluster	MI cluster
Geography (rural, suburban, urban)	0.10^§^	0.02^§^
Proportion of black population	0.02^§^	0.58^§^
Proportion population age ≥ 65 years	0.02^§^	0.19^§^
Proportion of single parent families	0.04^§^	0.08^§^
Proportion of owner occupied housing units	0.08^§^	0.02^§^
Median household income ($)	0.15^§^	0.03^§^
Proportion of population with < high school education	0.18^§^	0.00^§^
Proportion of married persons	0.20^§^	0.36
Average family size	0.34	0.27
Proportion of population living below poverty	0.41	0.00^§^
Median housing value (S)	0.41	0.00^§^
Gender	0.60^§^	0.04^§^
Proportion of population employed	0.67	0.00^§^

^§ ^Variables assessed in subsequent multivariable logistic regression model.

**Table 3:** Final logistic model showing socioeconomic and demographic predictors of high risk stroke mortality clusters

Variable	Coefficient	LRT* p-value	95% Confidence Interval
Constant	-6.036		-8.467, -3.605
Geography		0.17	
Rural	Referent		-
Suburban	1.299		-0.170, 2.769
Urban	1.351		-0.340, 3.042
Proportion of Blacks		0.02	
< 0.02	Referent		-
> 0.02 - ≤ 0.05	1.179		-0.127, 2.486
> 0.05 - ≤ 0.10	1.631		-0.095, 3.357
> 0.10	-0.629		-2.589, 1.35
Proportion of Pop with < High School education		0.02	
≤ 0.17	Referent		-
> 0.17 - ≤ 0.30	2.913		0.699, 5.127
> 0.30 - ≤ 0.37	3.022		0.740, 5.304
> 0.37	3.898		1.527, 6.268

*LRT (Likelihood ratio test) p-value = test of significance of each group of dummy variables (belonging to one categorical variable). Thus, this tests the statistical significance of the variable as a whole (all parameter estimates of the categories of variable in the model).

Goodness of fit tests showed no evidence (p = 0.389) that the model was not fitting the data well. The model had very high specificity (97.8%) (i.e. the ability to correctly predict no cluster given the neighborhood was not in a cluster). However, it had a relatively low (20%) sensitivity (i.e. the ability to predict being in a stroke cluster given that the neighborhood was truly in a cluster). The positive predictive value, or the probability of being in a cluster given the model predicted cluster, was 62.5%. The negative predictive value, or the probability of not being in a cluster given that the model predicted no cluster, was 87.4%. Overall, the model has a correct classification rate of 86.2%. There were a few outliers, with large positive residuals in the model. These neighborhoods were primarily urban, with the lowest proportion of population of blacks, and the lowest levels of population without high school education.

#### Myocardial infarction

The univariate associations of the socioeconomic and demographic variables of interest with the outcome of belonging to a high risk MI mortality cluster are presented in Table 2. Variables with significant associations, based on a liberal p-value = 0.20 were further assessed in the multivariable logistic model. The proportion of the neighborhood population of blacks was non-significant, but it was included in the analyses because disparities in MI risk and mortality by race have been reported in the literature [3,5,8,34]. The final model, based on the prescribed criteria for removal of variables, had a highly significant likelihood (p < 0,001) (Table 4). The proportion of the population with less than high school education, modeled as a continuous variable, was the strongest predictor of the odds of being in a MI cluster. Geography (p = 0.05) and gender (p = 0.03) were significant based on the likelihood ratio test of their respective dummy variables as a group. Suburban and urban neighborhoods had significantly higher odds of belonging to an MI cluster compared to rural neighborhoods. Neighborhoods with a higher proportion of males versus females also had higher odds of being in a cluster. The proportion of the population of black race was not significant (p = 0.1), but was included in the final model because it was an important confounder for both geography and gender such that its removal resulted in a more than 20% change for their coefficients. No interaction terms were significant at the p < 0.05 level.

**Table 4:** Final logistic model showing socioeconomic and demographic predictors of high risk myocardial infarction mortality clusters

Variable	Coefficient	LRT*	95% Confidence Interval
		p-value	
Constant	-6.541		-8.865, -4.220
Proportion of Pop with < High School education	14.562		8.963, 20.610
Geography		0.05	
Rural	Referent		-
Suburban	1.558		0.205, 2.911
Urban	1.544		-0.033, 3.122
Proportion of Blacks		0.14	
< 0.02	Referent		-
> 0.02 - ≤ 0.05	0.306		-0.844, 1.456
> 0.05 - ≤ 0.10	-0.991		-2.950, 0.968
> 0.10	-1.494		-3.231, 0.244
Gender		0.03	
Proportion of Male Population ≤ 0.50	Referent		-
Proportion of Male Population > 0.50	1.024		0.086, 1.962

LRT (Likelihood ratio test) p-value = test of significance of each group of dummy variables (belonging to one categorical variable). Thus, this tests the statistical significance of the variable as a whole (all parameter estimates of the categories of variable in the model).

Goodness of fit tests showed no evidence (p = 0.521) that the model was not fitting the data well. The model had very high specificity (90.2%). However, it had a relatively low (51.1%) sensitivity. The positive predictive value was 65.7% while the. negative predictive value was 83.3%. Overall, the model had a correct classification rate of 80%. There were only three neighborhoods that the model did not fit well. These were rural neighborhoods that had the most extreme high levels of the proportions of the population without high school education.

## Discussion

The results show that spatial patterns of high risk of stroke and MI exist in the study area. These findings are consistent with those from other studies that have reported that southern states like Tennessee [1,6,9,34,44], and specifically Appalachian counties [10,11,57], have excess risk of stroke and MI. The excess risk has mostly been attributed to variations in the distribution of stroke and MI risk factors such as race, socioeconomic status, geography (urban vs. rural), and prevalence of other chronic diseases, such as diabetes and hypertension [3,6,9,58]. However, other studies have reported that geographic disparities exist even after adjusting for variations in these risk factors [4,10,11,13]. The apparent inconsistency in the association between high risks of stroke/MI and risk factors at the state and county levels suggests that disparities may be due to more localized distributions of risk factors.

To our knowledge, this is the first study to investigate spatial patterns and clusters of stroke and MI risk to better understand observed disparities and identify specific health needs at the neighborhood level to aid population health planning. The results of the current study provide evidence that the risk of stroke and MI can be highly variable within a county and therefore studies that perform analyses at the county level fail to identify these disparities at lower (neighborhood) levels. For example, Knox and Hamblen counties are often reported to have lower risks of stroke and MI and are not considered economically distressed/disadvantaged when compared to other counties in the area [10,11]. However, it is evident from the findings here that a few neighborhoods in these counties have very high risks and are part of significant spatial clusters for stroke and MI. If analyses, research, and planning activities to address disparities in risk are conducted at county or higher levels as is often done, these spatial disparities within the counties would be missed. Therefore, neighborhoods would likely be erroneously ignored in programs geared towards addressing disparities in MI and stroke risk. The implication is that for health research and planning activities to be most effective, the focus must be on neighborhood level characteristics and specific needs to alleviate the variation seen at higher geographic levels.

Other studies have used multilevel analyses, including both neighborhood and individual characteristics, to describe disparities in MI risk for individuals [15-21]. One study, using data from the Atherosclerosis Risk in Communities Study, categorized neighborhoods (CTs) into tertiles by neighborhood median household income and found that greater incidence risk of MI was associated with living in lower income neighborhoods [38]. Diez Rouz, et al. (2001) also found that living in a disadvantaged neighborhood was associated with increased incidence of coronary heart disease, including MI, while adjusting for individual income, education, and occupation and defining neighborhoods as census block groups [18]. However, some differences in incidence remained between neighborhoods after adjusting for common socioeconomic factors. The failure of individual level risk factors to substantially explain risk at aggregated levels is a common finding in multilevel studies [45]. Some authors have suggested that neighborhood level socioeconomic variables capture information above and beyond the individual level, and so do not serve only as proxies for individual risk factors [21]. Similar to reports from other studies [16,21], we found that neighborhoods with a high proportion of the population with low education had higher stroke and MI risks. However, we did not find significant association between median household income and risk of MI or stroke. This is contrary to findings from previous studies [15,18,38,43] and is likely because these were individual level studies while ours is a population/group (neighborhood) level study. In addition to the level of education, the confounding identified between the geography (urban versus rural), race, and gender distribution of each neighborhood is potentially important to understanding how geographic disparities arise in the study area. The influence of neighborhood socioeconomic and social conditions on health may be related, in part, to availability and accessibility to health care services, the built environment and infrastructure (i.e. quality schools, recreational facilities, stores and restaurants with healthy foods), neighborhood based attitudes towards health and related behaviors (i.e. smoking, physical activity, and diet), and the degree of social support [14,20,59,60]. Since health planning is performed at the population level, identifying geographic disparities for neighborhoods can provide insight into the social conditions, structures, and mechanisms that influence health outcomes in the population to better provide effective population based education campaigns and prevention strategies. Thus, studies, such as this one, that investigate neighborhood level patterns in risk should be considered in addition to those multilevel studies that assess risk of individuals in neighborhoods to ensure community health resources, services, and other efforts are best targeted to the populations at greatest risk.

Although mortality data are useful and commonly used in epidemiological studies to assess health and its patterns, they are not without limitations. First, the accuracy of the cause of death given on a death certificate can be affected by errors made by physicians or in coding, differences in diagnostic criteria, issues arising when there are multiple causes of death, or errors in data entry [61]. Lloyd-Jones et al. (1998) reported that death certificates overrepresented coronary heart disease as cause of death, particularly for older populations, and cautioned that its use in etiologic studies could potentially lead to a bias towards the null value [62]. There is also concern that mortality data reflects past, rather than current, health needs. However, mortality is often the most commonly available data for observational, population-based studies since (in the US) it is freely available through organizations, like health departments and the Centers for Disease Control and Prevention [61]. Unfortunately, the mortality data in this study contained only decedent's residential address for geo-coding to the census tract level and gave no information on whether the address was a place other than a private home, such as nursing homes or prisons, thus limiting the ability to assess any effect such issues would have on the results of the study. However, we did identify to the best of our ability, the addresses known to be nursing homes and found that no more than 15 deaths occurred at any given address. Thus, we do not believe these issues would significantly affect the spatial patterns observed.

From a methodological standpoint, while neighborhood level analyses provide the advantage of better insight and understanding of health disparities and needs, they are not without limitations. Due to the small number problem, visualization of raw risks from areas with low population or small number of deaths can be misleading. In this study, this problem was overcome using SEB smoothing of risks that reduces noise associated with population heterogeneity and variance instability by borrowing strength from neighbors. While the removal of noise from low populations with unstable risks eases visual interpretation, it may possibly introduce artifacts into the map [24,63] and therefore these risks should only be used for visualization and not statistical analyses [64,65]. Additionally, many smoothing techniques, including the SEB used in this study, are prone to edge effects such that neighborhoods on the edges of the study area have fewer neighbors than those in the interior, so there is less information to borrow from neighbors in smoothing [23]. Thus the risks are shrunk toward a global instead of the local mean. Despite these disadvantages, spatial smoothing of risks minimizes erroneous visual interpretations associated with raw risks by reducing noise, making spatial patterns more evident, and reducing attention to outliers by focusing on the overall geographic pattern of the study area [23]. In this study, the smoothed risks did not change the raw pattern very much, except to make localized patterns more visually obvious for both stroke and MI. This result indicates that extreme values (very high and low risks) in the wide mortality risk range were composed of neighborhoods with stable risks, i.e. risks with low variance. Since the SEB has a larger impact on unstable risks and little to no impact on stable risks (i.e. those with low variances) [23,64], it is not unexpected that there were minimal differences between the raw (unsmoothed) and SEB risks.

The visual interpretation of spatial patterns can be strongly affected by the number and width of class intervals used to represent risk values [23,66]. To reduce this potential bias, it has been suggested that intervals should be based on the overall shape of the distribution and not statistical frequency [66]. Thus, this study employed the Jenks, or natural breaks, classification method which defines intervals based on the natural distribution of breaks or groupings in the data [67]. The visualization of spatial patterns of disease is an important component in identifying geographic disparities. However, it is standard epidemiology practice not to rely on one's visual interpretation of a map of disease risks to differentiate significant spatial clusters from what may seem to be a cluster visually but is not statistically significant [24,65]. Furthermore, interpretations of spatial patterns from visual investigations become even more difficult when the population is heterogeneously distributed throughout the study area, resulting in differences in variances of disease risks across different areas in the map. Thus, statistical comparisons are needed to identify areas where statistically significant clusters of stroke and MI mortality exist, while taking into account population distribution, to better understand disease disparities. This explains the need to use SEB risk maps as well as spatial scan statistics to identify significant high risk spatial clusters. Moreover, other studies have also indicated that interpreting the results of cluster detection along with the spatial distribution of risk, especially with Bayesian smoothing, can strengthen findings of spatial analysis [68-70].

Spatial scan statistics were used to identify and assess the statistical significance of areas with high risk of stroke and MI clusters. This methodology, implemented in SaTScan 8.0 [71], has many advantages over other cluster detection methods: it corrects for multiple comparisons, adjusts for population heterogeneity in the study area, identifies clusters without *a priori *specification of their suspected location or size and thus limits pre-selection bias, and allows for adjustment for covariates [54,72]. Using visualization of spatial patterns of SEB smoothed risk in conjunction with the results of spatial scan statistics in this study, the neighborhoods with the highest risks were consistent and easy to identify. Detection of spatial clusters of disease allows health planners to effectively identify and plan for the specific characteristics and health needs of the populations with the highest risks of disease [68,69]. For instance, median levels of stroke and MI mortality risk were observed for Knox County in the smoothed risk maps, but cluster detection highlighted just a few neighborhoods with statistically significant higher risk than surrounding neighborhoods in the county. The implication is that health planning and programs can be focused to specific neighborhoods of high risk to better meet their health needs instead of using a one-size-fits-all strategy for all neighborhoods within a county. Thus, neighborhood level analysis allows limited resources and efforts to be targeted to the highest risk communities [68].

## Conclusion

Spatial clusters of high mortality risks were identified at the neighborhood level, indicating disparities in risk of death from MI and stroke within counties of the study area. The implication is that, from a needs-based health planning standpoint, a neighborhood level approach is important to ensure that resources and efforts are targeted to the populations most in need. This study also demonstrated that the use of spatial statistics, cluster detection methods, and GIS can aid health planners in appropriately assessing and identifying spatial disparities in risk in populations so as to better guide evidence-based health planning decisions.

## List of Abbreviations Used

CDC: Centers for Disease Control and Prevention; CT: census tracts; ICD: International classification of diseases; GIS: geographic information systems; MI: myocardial infarction; SEB- spatial empirical Bayes; TN: Tennessee; US: United States.

## Competing interests

The authors declare that they have no competing interests.

## Authors' contributions

AP was involved in data acquisition, analyses, and interpretation, as well as preparation of the manuscript. TA was involved in data acquisition and review of the manuscript. AO conceived the research idea and was involved in data acquisition, study design, interpretation of results, as well as extensive editing of the manuscript. All authors certify that they have participated sufficiently in the research to believe in its overall validity and have read and approved the final manuscript.

## Pre-publication history

The pre-publication history for this paper can be accessed here:

http://www.biomedcentral.com/1471-2458/11/644/prepub
